# Homœopathy *vs.* Intermittent Fever

**Published:** 1888-01

**Authors:** Samuel Long

**Affiliations:** New Brunswick, N. J.


### HOMŒOPATHY *vs.* INTERMITTENT FEVER.

(Read before the Homœopathic Medical Society of New Jersey, Oct., 1887.)

In selecting Homœopathy as opposed to intermittent fever for my subject, I will not say anything new or original, but do what I can to further the cause which I have espoused; and in selecting intermittent fever I have chosen a form of malaria which has caused many of our school to waver and stumble into eclecticism and mongrelism.

Homœopathy is a system of scientific medicine founded and practiced by Samuel Hahnemann, who graduated as an allopathic physician at Erlangen, Germany, in the year 1779. He practiced as an allopath, or "regular," for a dozen years, and at the ripe age of thirty-five became disgusted with physic, and "considered all ordinary medication worse than nothing, not only no good, but a positively hurtful art."

He abandoned the practice for a few years, during which time he devoted himself to literary work and medical investigations, the practical outcome of which was the discovery of the only true and universal law of cure, "*similia similibus curantur.*" Had he left merely this law as a legacy for suffering humanity, he would be immortal; he not only did this, but added his book, the *Organon*, which is an exhaustless mine of knowledge of the law, together with the rules and principles regulating it.

By his efforts medicine became a science, and like all sciences, has developed and enlarged without changing its original principles; and no advance in practical medicine has been made without using his inductive method. Since Hahnemann's time, we to-day have the lives and works of many of his illustrious followers, who developed these rules and principles and gave us their testimony of their worth.

Now, this same Homœopathy had its beginning and rise with intermittent fever; I refer to the time when Hahnemann in translating Cullen's *Matéria Medica*, added the foot-note concerning Cinchona. He afterward showed that "Peruvian bark" would not cure all cases of "chills and fever," but only those to which it was homœopathic, and when we read of the country and districts, in which he practiced, infected with pernicious intermittents, and know that he was successful, is it not a strange and sad fact that those who assume to be his followers in treating this same disease ignore his teachings and forsake him? For I believe that this same fever is the subtle temptation that tries the character of every one of our school, and causes many to take the first step toward eclecticism! When I read of and consider intermittent fever, I am often impressed with this coincidence (if you choose to call it such), that the very disease which called this mode of practice into the world, is now causing physicians to be untrue to their profession and their patients to live a miserable existence.

Perhaps the assertion "that intermittent fever patients lead a miserable existence" may be questioned, but if the history be traced of all chronic cases that appeal for the cure of neuralgias, enlarged liver and spleen, dyspepsia and consumption, laryngitis, bronchitis, and even phthisis, deafness and buzzing in ears, it will frequently be found that they have their origin in the suppression of this fever.

It is unnecessary at this time to give its history and symptoms, suffice it to say that all authors are agreed as to its being universal in its manifestations—we find it in the mountain range as well as in the valley; along the seacoast as well as inland; and that every individual, young or old, may be liable to its attack. We believe the law of Homœopathy equally universal and applicable to every case.

Having in this very brief way called your attention to this subject, let us next inquire what should be our attitude when called upon to treat this disease.

Every one is, to a great degree, just what his beginnings and environments have made him, and every physician is largely what his preceptor* and college have made *him*; fortunate is he who has had a right beginning, and is controlled by truth and and principle! As a student of homœopathic medicine I entered the office of, and carefully followed for over two years the prac-

* The influence of a preceptor on the mind of a student, for good or for evil, is very great. Lucky are those who fall into such good hands as did Dr. Long; the preceptor he refers to has turned out some of the very best physicians our school can now boast of; all honor to him.—Eds.

tice of, a firm and true disciple of Hahnemann; I became intimately acquainted with his patients, witnessed the results of his prescriptions, and, on every hand, heard him referred to with honor and respect.

A few months after my graduation from Hahnemann Medical College, of Philadelphia, March, 1873, I selected New Brunswick, New Jersey, a city of twenty thousand inhabitants, on the west bank of Raritan River, given over entirely, as to medical matters, to allopathy and ecclecticism.

"I was a perfect stranger in a strange land," and, as I afterward learned, in the most conservative place in all Jersey! Very few know the trials and difficulties of the first ten years of my practice; but you can picture them when I say that Hahnemann and the *Organon* were the controlling motives of my practice, and that I have conscientiously followed them to this day. I claim that any one who employs me wants Homœopathy, and that its demands must be recognized first; I am only a trustee, so to speak, and he who is unwilling to abide by the application of these principles must go elsewhere; my first duty is to Homœopathy, my own welfare and interests come second—and thus have I ever endeavored to create a sentiment for this particular mode of practice in this community.

I know that this is unpopular for a season, and difficult to follow; but it has this happy effect, your patients know what to expect, you have control of the case, and no interference with busy nurses, local treatment, and tonics.

And thus I have learned to accept our materia medica as alone sufficient for the cure of this and all other diseases, and concerning which Dr. P. P. Wells in No. 9, Vol. IV, HOMŒOPATHIC PHYSICIAN, says: "What is it in the drug agent that makes men sick? This power which cures—what is it? A right answer to this and a right understanding of its nature may be set down as indispensable to test practical results from its clinical use. We have said, Homœopathy 'deals not with the material mass of the drug, but with its developed and liberated spirit.' By this we mean it deals with that something in the drug which has power to make human organs and functions sick. If there be an objection to the use of the word *spirit*, we will not make a quarrel with any man for the word who will bring us one which better expresses the nature of that power which has been planted in and associated with the drug, and which we have so abundant evidence is something distinct from the material substance of the drug."

And thus it is my conviction, after making use of nothing but potentized remedies for the cure of all diseases during all these years, that the rules and principles regulating and controlling body and soul are "similar," as illustrated in that excellent work recently published by Professor Drummond: *The Natural Law in the Spiritual World.*

SAMUEL LONG, M. D.

NEW BRUNSWICK, N. J., October, 1887.

